# Fecal *Filobasidium* Is Associated with Clinical Remission and Endoscopic Response following Fecal Microbiota Transplantation in Mild-to-Moderate Ulcerative Colitis

**DOI:** 10.3390/microorganisms10040737

**Published:** 2022-03-29

**Authors:** Isabelle A. M. van Thiel, Shafaque Rahman, Theodorus B. M. Hakvoort, Mark Davids, Caroline Verseijden, Patricia H. P. van Hamersveld, Mèlanie V. Bénard, Maarten H. Lodders, Teun Boekhout, René M. van den Wijngaard, Sigrid E. M. Heinsbroek, Cyriel Y. Ponsioen, Wouter J. de Jonge

**Affiliations:** 1 Tytgat Institute for Liver and Intestinal Research, Amsterdam University Medical Centers, Location AMC, University of Amsterdam, Meibergdreef 9, 1105 AZ Amsterdam, The Netherlands; i.a.vanthiel@amsterdamumc.nl (I.A.M.v.T.); s.rahman@amsterdamumc.nl (S.R.); t.hakvoort@amsterdamumc.nl (T.B.M.H.); c.verseijden@amsterdamumc.nl (C.V.); h.p.vanhamersveld@amsterdamumc.nl (P.H.P.v.H.); m.h.lodders@gmail.com (M.H.L.); r.vandenwijngaard@amsterdamumc.nl (R.M.v.d.W.); s.e.heinsbroek@amsterdamumc.nl (S.E.M.H.); 2 Amsterdam Gastroenterology, Endocrinology, Metabolism (AGEM), 1105 AZ Amsterdam, The Netherlands; 3 Microbiota Center Amsterdam (MiCA), Meibergdreef 9, 1105 AZ Amsterdam, The Netherlands; m.davids@amsterdamumc.nl; 4 Department of Vascular Medicine, Amsterdam University Medical Centers, Location AMC, University of Amsterdam, Meibergdreef 9, 1105 AZ Amsterdam, The Netherlands; 5 Department of Gastroenterology and Hepatology, Amsterdam University Medical Centers, Location AMC, University of Amsterdam, Meibergdreef 9, 1105 AZ Amsterdam, The Netherlands; m.v.benard@amsterdamumc.nl (M.V.B.); c.y.ponsioen@amsterdamumc.nl (C.Y.P.); 6 Westerdijk Fungal Biodiversity Institute, Uppsalalaan 8, 3584 CT Utrecht, The Netherlands; t.boekhout@wi.knaw.nl; 7 Institute of Biodiversity and Ecosystem Dynamics (IBED), University of Amsterdam, Sciencepark 904, 1098 XH Amsterdam, The Netherlands; 8 Department of Surgery, University Hospital Bonn, 53113 Bonn, Germany

**Keywords:** fecal microbiota transfer, *Filobasidium*, *Candida*, ulcerative colitis, macrophages

## Abstract

Fecal microbiota transplantation (FMT) has the potential to restore (bacterial and fungal) microbial imbalance in ulcerative colitis (UC) patients and contribute to disease remission. Here, we aimed to identify fecal fungal species associated with the induction of clinical remission and endoscopic response to FMT for patients with mild-to-moderate ulcerative colitis. We analyzed the internal transcribed spacer 1 (ITS1)-based mycobiota composition in fecal samples from patients (*n* = 31) and donors (*n* = 7) that participated previously in a double-blinded randomized control trial evaluating the efficacy of two infusions of donor FMT compared with autologous FMT. The abundance of the yeast genus *Filobasidium* in fecal material used for transplantation was shown to correlate with clinical remission following FMT, irrespective of its presence in the material of donor or autologous fecal microbiota transfer. The amplified sequence variants within the genus *Filobasidium* most closely resembled *Filobasidium magnum*. Monocyte-derived macrophages and HT29 epithelial cells were stimulated with fungal species. Especially *Filobasidium floriforme* elicited an IL10 response in monocyte-derived macrophages, along with secretion of other cytokines following stimulation with other *Filobasidium* species. No effect of *Filobasidium* spp. was seen on epithelial wound healing in scratch assays. In conclusion, the enriched presence of *Filobasidium* spp. in donor feces is associated with the positive response to FMT for patients with UC and hence it may serve as a predictive fungal biomarker for successful FMT.

## 1. Introduction

An aberrant response to luminal gut microbes has been associated with many diseases, including Crohn’s disease (CD) and ulcerative colitis (UC): both inflammatory bowel diseases (IBD) [1,2]. While the bacterial contribution has been studied extensively [3], the fungal counterpart has only gained attention in recent studies. For example, there are reports concerning alterations of luminal gut fungi (i.e., mycobiota) diversity and composition in UC patients [4,5,6,7]. In addition, several individual fungal species were recently associated with the severity of (experimental) colitis [8,9], thereby confirming a role for the gut mycobiota in intestinal inflammation. Restoration of microbial communities to resemble that of a healthy individual is considered beneficial for resolving intestinal inflammation or maintaining remission [10]. As such, several randomized-controlled studies have explored the potential of fecal microbiota transplantation (FMT) as a means to improving gut microbial populations in IBD, but unfortunately the efficacy of these complex and invasive procedures remains relatively low [10] and a stratification strategy of FMT candidate patients is warranted. To increase efficacy, donor material may be derived from selected suitable candidates for transplantation based on earlier associations with response, e.g., that of so-called superdonors [11], known to induce remission. On the other hand, the microbial composition of the acceptors of FMT may also be a contributing factor to the success of this procedure. The role of the mycobiome in the success of FMT has not been clarified. It was recently shown that a high abundance of the genus *Candida* in acceptors prior to FMT was associated with a positive response to transplantation with allogenic feces [6]. In the current study we aimed to determine fungal markers both in patient and donor fecal material that associate with a response to FMT for patients with mild to moderate UC.

In order to answer this research question, we analyzed samples of the previously performed TURN trial (Transplantation of Feces in Ulcerative Colitis, Returning Nature’s Homeostasis). This study consisted of a randomized, double-blind FMT intervention for patients with mild-to-moderate UC [12]. Patients received two duodenal infusions of feces from either healthy donors (FMT-D) or autologous material (FMT-A) with an interval of three weeks. Response to FMT was defined as clinical remission (simple clinical colitis activity index ≤ 2) and endoscopic response (Mayo score ≥ 1-point decrease) at 12 weeks after the first intervention, which was not significantly higher for FMT with donor feces (FMT-D; 41.2% (7 of 17 (per protocol analysis))) than for FMT with autologous feces (FMT-A; 20% (5 of 20 (per protocol analysis))). While bacterial profiles were previously determined, these merely indicated a change among FMT-D responders towards their respective donor microbiota profile [12]. However, no bacterial signature or differentially abundant genera were observed at baseline between positive responders and non-responders in the original study [12,13]. In the current study, we assessed fecal mycobiota profiles of patients and donors enrolled in the previously performed TURN trial. In line with previous findings, elevated fecal abundance of *Candida* spp. in acceptors of FMT-D was associated with responsiveness in this cohort. In addition, we found that the abundance of the genus *Filobasidium* in fecal material used for transplantation, independent of fecal material being derived from FMT-D or FMT-A, associates with responsiveness to FMT for UC. Selected *Filobasidium* species also elicited the release of IL10 and other cytokines in macrophages, suggesting that this species elicits an inflammatory response. Based on this study, we suggest assessing gut mycobiota profiles in addition to bacterial microbiota profiles as indicators for FMT therapeutic success.

## 2. Materials and Methods

### 2.1. Patients and Donors

Patient recruitment and fecal microbiota transfer protocol recruitment were described previously [12]. Patients did not receive antibiotics up to six weeks prior to inclusion. In the original study, 37 patients were treated per protocol. Six of these patients received their second FMT from a different donor because of the unavailability of the original donor. In the current study, we only use a restricted set of single-donor FMTs (*n* = 11 FMT-D, *n* = 20 FMT-A) to rule out the effect of multi-donor FMTs. Response (i.e., clinical remission (simple clinical colitis activity index ≤ 2) and endoscopic improvement (Mayo score ≥ 1-point decrease) at 12 weeks after the first FMT [12]) was achieved for 9 of these patients (Table 1).

Fecal material from 7 donors was used for the treatment of these patients. Donors were allowed to donate fecal material for multiple patients. Four donors were anonymous volunteers, and three donors were a spouse or friend of the respective patient. The median age of the donors was 29 years (SD = 10) and 71% were male (*n* = 5). Donors were not allowed to have used antibiotics within eight weeks before screening.

In total, we obtained 48 patient samples (*n* = 24 at baseline, *n* = 24 samples at week 12), and 10 donor samples (at baseline) for analysis.

### 2.2. Fecal DNA Isolation and Internal Transcribed Spacer 1 (ITS1) Sequencing

Fecal DNA was isolated previously in light of the original trial report [12]. Generation of ITS1 amplicons libraries and subsequent sequencing were performed as previously described [14]. In brief, fecal DNA was subjected to a two-step polymerase chain reaction (PCR). In the first reaction, ITS1-regions were amplified, and overhang was created for the Illumina Nextera platform. During the next reaction, Illumina sequencing adapters were introduced, resulting in 200–700 base pair amplicons. Samples were mixed based on DNA concentrations as determined by Qubit Fluorometric quantitation, and equal amounts were sequenced using an Illumina MiSeq machine (600V3, paired end; San Diego, CA, United States).

USEARCH was used to merge resulting reads. One sample (baseline, non-responder, FMT-A) did not have sufficient reads and was omitted for downstream analysis. Reads that were longer than the sequenced range, and thus could not be merged, were concatenated. Amplified sequence variants (ASV) were inferred using UNOISE3 on per sample basis. ASV taxonomy was determined using the Bayesian classifier [15] and the UNITE database (V02.02.2019, [16]). ASV abundance was determined by mapping the merged and concatenated reads against the collectively inferred ASV set.

Mycobiota sequencing data were analyzed and visualized in R (v4.1.1) and compiled into a phyloseq object (v1.36.0, [17]). Phylogenetic tree of *Filobasidium* spp. and *Cryptococcus* spp. was constructed using reference data from selected references strain from the UNITE database (v02.02.2019, [16]), multi-locus sequence alignment (package msa, v1.24.0, [18]) and visualized using ggtree (v3.0.4, [19]). All raw sequencing data have been submitted to the ENA database under accession number PRJEB45955. Statistical analysis of mycobiota sequencing analysis is described under ‘Section 2.8 Statistical analysis’.

### 2.3. Fungal Strains

Three species of the genus *Filobasidium* (*Filobasidium magnum* (CBS 140), *F. floriforme* (CBS 6241), *F. uniguttulatum* (CBS 1730)) were obtained from Westerdijk Fungal Biodiversity Institute (Utrecht, the Netherlands). *Candida albicans* (CBS 16801) was obtained from the feces of a healthy volunteer that gave informed consent [20]. All yeast cultures were inoculated at high density and grown aerobically overnight in Sabouraud Dextrose Broth (Sigma Aldrich, Zwijndrecht, The Netherlands) at 24 °C.

In stimulation experiments, the fixed yeast cells were used. Yeast cells were washed three times in phosphate-buffered saline (PBS; Fresenius Kabi, Huis Ter Heide, The Netherlands) and fixed in 4% paraformaldehyde in PBS for 1 h at room temperature. Yeast cells were washed again, resuspended in PBS and stored at −20 °C until use. Fixation was confirmed by lack of growth when culturing fungal stock solution on Sabouraud Dextrose Agar (Sigma Aldrich) and assessing growth after 7 days incubation at 37 °C. Before stimulation experiments, cells were counted and subsequently diluted in appropriate cell culture medium.

### 2.4. Monocyte-Derived Macrophages Stimulations

Monocytes were isolated from buffycoats obtained from healthy volunteers (Sanquin, Amsterdam, The Netherlands). At first, peripheral blood mononuclear cells (PBMCs) were isolated by Ficoll density gradient (VWR International BV, Amsterdam, The Netherlands). Monocytes were isolated by hyper-osmotic Percoll (VWR International BV) density gradient centrifugation as described previously [21]. Cells were maintained in Iscove’s Modified Dulbecco’s Medium (IMDM) supplemented with 2 mM L-glutamine and 25 mM HEPES (Thermo Fisher Scientific, Bleiswijk, The Netherlands), 10% (*v*/*v*) heat-inactivated fetal bovine serum (FBS; Serana, Pessin, Germany) and 100 U/mL penicillin and 100 μg/mL streptomycin (Fisher Emergo B.V, Landsmeer, The Netherlands) at 37 °C and with 5% CO_2_. Cells were plated at a concentration of 1 × 10^6^ cells/mL in a 12-well culture plate (VWR International BV), and 1 × 10^5^ cell /100 µL in a 96-well cell culture plate (Corning Life Sciences BV, Amsterdam, The Netherlands). Isolated monocytes were stimulated with 20 ng/mL of human macrophage colony-stimulating factor (hMCSF; Peprotech, Rocky Hill, NJ, USA) for 3 days. Cells were washed once with warm PBS and subsequently polarized to M1 with 50 ng/mL of interferon gamma (IFNγ; Peprotech) and M2 with 40 ng/mL of IL4 (Peprotech), respectively for 3 additional days. Cells polarized into M0 macrophages were maintained in complete medium without additional cytokines.

In a selection of experiments, the polarized macrophages were pre-stimulated for 1 h with 100 ng/mL lipopolysachcharide (LPS; Sigma Aldrich), followed by a single wash in complete IMDM. Macrophages were stimulated with fixed yeast cells in 1:1 ratio to macrophages. Cells were harvested either after 4 h for gene expression analysis, or supernatant was harvested after 24 h for cytokine release analysis.

### 2.5. Epithelial Scratch Assays and Stimulation Experiments

HT29 colon carcinoma epithelial cells were maintained in Dulbecco’s Modified Eagle Medium (DMEM; Lonza) with 10% FBS (Serana), 2 mM L-glutamine (Lonza), and 100 U/mL penicillin and 100 μg/mL streptomycin (Fisher Emergo) at 37 °C and 5% CO_2_. Cell cultures were routinely screened for mycoplasma infection, which did not occur during the course of experiments. Five days prior to infection, HT29 cells were seeded at a density of 3 × 10^5^ cells/mL in a 24-wells culture plate (VWR International BV). An amount of 10 ng/mL tumor necrosis factor alpha (TNFα; PeproTech) was used to mimic inflammatory conditions. Yeasts were added at a final concentration of 1 × 10^6^ cells/mL. Cells were harvested either after 4 h for gene expression analysis, or supernatant was harvested after 24 h for cytokine release analysis.

Scratch healing assays were performed as previously described [22]. In brief, HT29 cells were seeded at a density of 3 × 10^5^ cells/mL in a 12 wells plate (VWR International BV). Cells were grown to confluency, which occurred after 5 or 6 days. Scratches were made using a 200 μL pipette tip (Greiner Bio-One BV, Alphen aan den Rijn, The Netherlands). Immediately thereafter, yeasts were added at a final concentration of 1 × 10^6^ cells/mL and/or TNFα at a final concentration of 10 ng/mL. Cell migration was filmed overnight using a DMi8 inverted microscope (Leica, Wetzlar, Germany) with a humidified culture chamber maintained at 37 °C and 5% CO_2_. Conditions were tested in duplicate and repeated three times. Each scratch was analyzed on two positions to reduce technical variation. Images were analyzed using ImageJ software (version 1.50i, [23]).

### 2.6. Cytokine Determinations (CBA, ELISA)

Concentrations of TNFα, IL6 (stimulated with yeasts cells without LPS) and IL10, IL1β (for all stimulations) in cell culture supernatants were analyzed by cytometric bead array (CBA, BD Bioscience, Vianen, The Netherlands) with a 3-fold sample dilution in PBS. TNFα and IL6 (pre-stimulation with LPS followed by yeast cell stimulations) and IL8 concentrations were determined by the sandwich enzyme-linked immunosorbent assay (ELISA; R&D Systems, Abingdon, UK) according to manufacturer’s protocol. Optical density was measured using a Synergy HT plate reader (BioTEK, Beun de Ronde, Abcoude, The Netherlands).

### 2.7. RNA Isolation, First-Strand Synthesis, Qualitative PCR

RNA isolation was performed using Bioline ISOLATE II Mini kit (GC Biotech, Alphen aan den Rijn, The Netherlands), according to manufacturer’s protocol. RNA concentrations were determined using Nanodrop ND1000 (Thermo Fisher Scientific). First-strand synthesis was performed using 2.5 ng/µL random hexamer primers (Promega, Leiden, The Netherlands), 10 µM oligo dT primers, 1 mM deoxyribonucleotide triphosphate (dNTPs), 1× RT-buffer, 1 U/µL Ribolock RNAse Inhibitor and 5 U/µL RevertAid Transcriptase (all from Thermo Fisher Scientific) in a total volume of 20 μL. Gene expression was performed using SensiFAST SYBR No-ROX (GC Biotech BV, Waddinxveen, The Netherlands) on a CFX96 machine (Bio-Rad Laboratories BV, Lunteren, The Netherlands). Most stable reference genes were determined by geNorm analysis [24], being *PPIA* and *PSMB6* for monocyte-derived macrophages and *HPRT*, *GAPDH* for HT29 cells. Gene expression of targets genes was determined using LinRegPCR software [25]. Primer sequences (Sigma Aldrich) are listed in Table 2.

### 2.8. Statistical Analysis

Patient baseline characteristics are reported as mean (SD) for normally distributed continuous data, and as median (IQR) for non-parametrically continuous data. Categorical data were displayed as frequencies (percentages). Data were analyzed using IBM SPSS Statistics (version 26; Chicago, IL, United States). Patient baseline data were not tested for significance in line with The Strengthening the Reporting of Observational Studies in Epidemiology (STROBE) guidelines [26].

Mycobiota data were visualized using ggplot2 (v3.3.5, [27]). For analysis of *Candida* spp. abundance, Basidiomycota to Ascomycota ratio, composition analysis, and *Filobasidium* spp. abundance, fungal counts were rarefied to the lowest sample sum, being 52,786 reads. Differentially abundant genera were determined using the DESeq2 package (v1.32.0, [28]), for which a false discovery rate (FDR) below 0.05 was considered significant. Linear mixed effects models (package lme4, v1.1-27.1, [29]) were used to test significant of Basidiomycota to Ascomycota ratios and *Candida* spp. and *Filobasidium* spp. abundances. Wilcoxon signed-rank test was used in case of comparison of two groups, and Kruskal–Wallis and Dunn’s multiple comparisons test were used for multiple groups. Compositional differences based on Bray–Curtis dissimilarity (phyloseq) were tested using permutation multivariate analysis of variance (PERMANOVA, package vegan (v2.5-7, [30])). Compositional shift over time were tested using a stratified PERMANOVA.

In vitro experiments are considered non-parametrically distributed and are analyzed by Kruskal–Wallis test and Dunn’s post hoc test. Data of in vitro experiments are presented as median and separate data points. GraphPad Prism (version 9.1.0; San Diego, CA, United States) was used for data visualization and statistical tests. *p* values below 0.05 are considered significant.

### 2.9. Ethical Considerations

All participants in the FMT study gave their written informed consent before the fecal samples were collected. This study was approved by the Medical Ethics Committee at Academic Medical Hospital, Amsterdam and was registered on ClinicalTrials.gov (NTC01650038). For buffy coats of healthy donors, informed consents were received from participants and approved by Amsterdam UMC Institutional Review Board.

## 3. Results

### 3.1. Elevated Filobasidium spp. Abundance in Transplanted Fecal Material Associates with Positive Response to Fecal Microbiota Transfer

**Figure 1:**
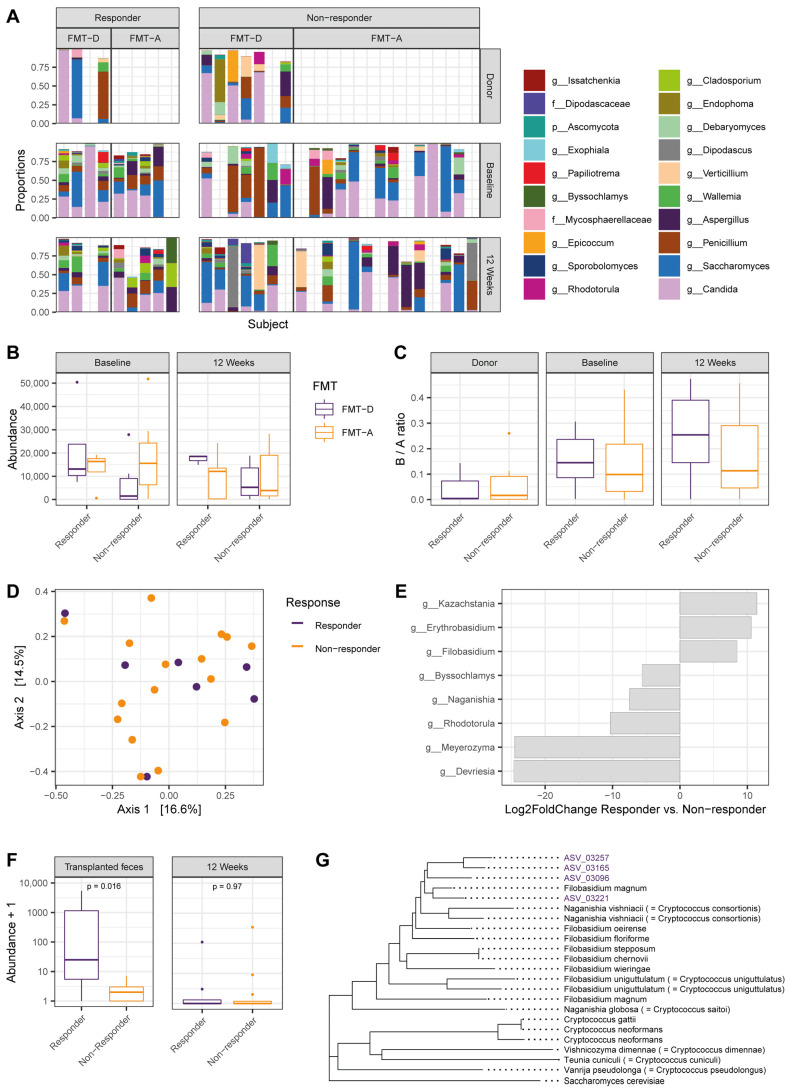
*Filobasidium* spp. abundance in donor material is associated with positive response to fecal microbiota transfer (FMT). (**A**) Internal transcribed spacer 1 (ITS1)-based mycobiome composition plot showing abundance of on genus level, with subjects stratified for response to FMT with donor feces (FMT-D) or with autologous feces (FMT-A). Each column represents one patient sample set. Characters in taxa list indicate taxonomic rank (g, genus; f, family). Donor samples are only displayed for FMT-D as FMT-A uses baseline fecal material. FMT-D, fecal microbiota transfer using donor feces; FMT-A, fecal microbiota transfer using autologous feces. (**B**) Abundance of the genus *Candida* in donors and patient samples at baseline or at 12-weeks follow-up. FMT-D responders vs. FMT-D non-responders at baseline, *p* = 0.014 (Kruskal–Wallis). (**C**) Basidiomycota to Ascomycota ratio. (**D**) Mycobiome composition of fecal material for transplantation (both FMT-A and FMT-D) based on Bray–Curtis dissimilarities is not different between responders and non-responders. (**E**) Significant differentially abundant genera in fecal material for transplantation (p_adj_ < 0.05). Bar length indicates Log2FoldChange. (**F**) Abundance of the genus *Filobasidium* spp. in fecal matter for FMT and at 12 weeks after the first FMT. (**G**) Phylogenetic tree of *Filobasidium* and *Cryptococcus* clades. Names in brackets are former names. Purple taxa indicated with ‘ASV’ are the four most abundant amplicon sequence variants (ASVs) within the genus *Filobasidium* in this cohort. Length of each tip indicates genetic similarity between neighboring taxa.

In order to associate the fecal mycobiota profiles of patients undergoing FMT with responsiveness to this intervention, we analyzed samples of a previous trial in ulcerative colitis. Since FMT-A also involves manipulation and infusion of fecal material and thereby reduced disease activity, we stratified patients for response rather than FMT type. As such, we will refer to the term ‘fecal material for transplantation’ as fecal matter to be infused into a patient irrespective of whether this material is autologous or obtained from a donor. ITS-1 sequencing was performed on a total of 48 samples obtained from 31 single-donor FMTs (*n* = 22 non-responders, *n* = 9 responders) and the respective donors. The genus *Candida* dominated the mycobiota profile in the majority of samples (Figure 1A). A high abundance of the yeast genus *Candida* in acceptors of FMT-D was previously associated with responsiveness to FMT-D [6], and this association was also present in the current cohort (Figure 1B, *p* = 0.019, Kruskal–Wallis and Dunn’s post hoc test). Furthermore, at phylum level, an elevated Basidiomycota to Ascomycota (B/A) ratio was observed in patients with IBD as compared to healthy donors, especially in those with active disease [4]. In the current cohort, the B/A ratio of patients with UC was higher than that of healthy donors, although this did not reach significance in this sample size (Figure 1C, *p* = 0.059). When assessing the mycobiota composition of all fecal material for transplantation based on Bray–Curtis dissimilarities, no differences were found between infused material of responders and non-responders (Figure 1D, *p* = 0.979, PERMANOVA). In addition, the overall mycobiota composition of both responders and non-responders did not change significantly over time. In conclusion, the fungal mycobiota composition of transplanted fecal material does not differ between responders and non-responders and patients’ mycobiota profiles do not undergo structural changes during treatment.

Since the general mycobiota structure was not associated with response to FMT, differentially abundant genera in the transplanted material samples were assessed next to evaluate whether specific fungal taxa may associate with positive outcome following FMT. Eight genera were differentially abundant (Figure 1E, p_adj_ < 0.05), of which the three genera *Kazachstania*, *Erythrobasidium*, and *Filobasidium* were more abundant in transplanted fecal matter of responders. While *Kazachstania* sp. [31] and *Rhodotorula* sp. [32,33] were previously discussed in rodent models of IBD, only *Filobasidium uniguttulatum* was previously found in association with non-inflamed mucosa of patients with active Crohn’s disease [34]. The abundance of the genus *Filobasidium* was significantly higher in fecal material for transplantation to responders than non-responders (*p* = 0.016, Figure 1F), at baseline but not at 12 weeks after the first FMT. The four most abundant ASVs of the genus *Filobasidium* within our dataset (ASV_03257, ASV_03165, ASV_03096, ASV_03221) all positively associated with response to FMT.

Notably, identification at the species level is commonly difficult at the resolution of ITS1 sequencing to provide this level of discrimination. Based on the phylogenetic tree of the *Filobasidium* and *Cryptococcus* clades, the ASVs most closely resemble *Filobasidium magnum* (Figure 1G). Taken together, an elevated abundance of the genus *Filobasidium* in transplanted fecal material, independent of FMT type, is associated with a clinical and endoscopic response to FMT.

### 3.2. Filobasidium spp. Stimulation of Monocyte-Derived Macrophages Elicits Cytokine Responses

**Figure 2:**
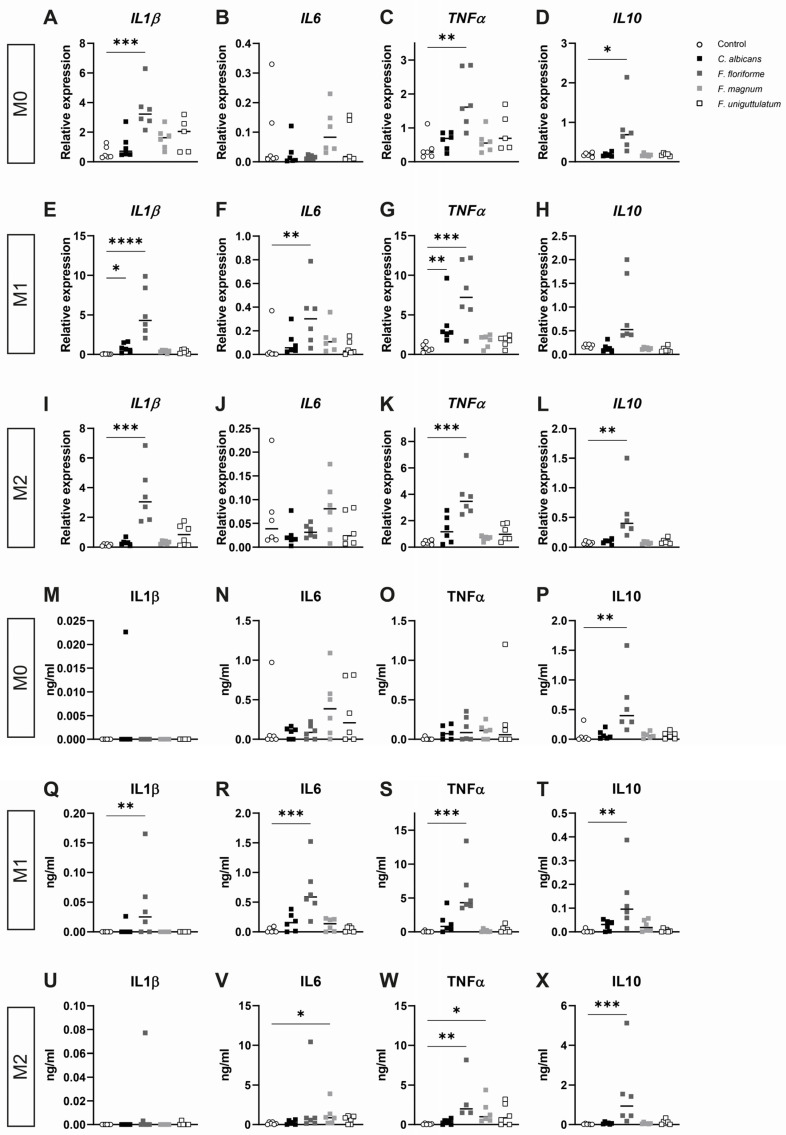
Stimulation of monocyte-derived macrophages to *Filobasidium* spp. results in cytokine response. (**A**–**L**) Relative expression levels of cytokines IL1β, IL6, TNFα, and IL10 across M0, M1, and M2 polarized monocyte derived macrophages ((**A**–**D**), (**E**–**H**), (**I**–**L**), respectively) after 4 h stimulation with yeast species (*n* = 5–6 individuals). mRNA expressions are normalized against housekeeping genes, *PPIA* and *PSMB6*. (**M**–**X**) Released cytokines (IL1β, IL6, TNFα, IL10) by monocyte-derived macrophages skewed into M0, M1, or M2 phenotype ((**M**–**P**), (**Q**–**T**), (**U**–**X**), respectively) and stimulated with yeasts for 24 h (*n* = 4–6 individuals). Data represented as median and separate data points. * *p* < 0.05; ** *p* < 0.01; *** *p* < 0.005; **** *p* < 0.001.

As abundance of the genus *Filobasidium* positively associated with beneficial response to FMT in the current cohort and was previously found in association with non-inflamed mucosa [34], we next hypothesized that *Filobasidium* spp. may have a functional relation with FMT therapeutic outcome through interaction with intestinal immune cells. Specifically, macrophages possess a broad spectrum of modulatory effector functions, including (regulation of) inflammatory processes [35], tissue healing [36], and even maintaining epithelial function by controlling fungal metabolite uptake [37]. To test the cellular effects of *Filobasidium* spp., peripheral blood monocyte-derived macrophages were first polarized into either M0, M1, or M2 phenotype. Cells were stimulated with either one of three species of the genus *Filobasidium*: *Filobasidium uniguttulatum* was previously described, *Filobasidium magnum* is the most likely species based on the current sequencing data, and *Filobasidium floriforme* is most compatible with human physiology as this species is able to withstand temperatures of 37 °C. *C. albicans* was used as reference for macrophage response given the known opportunistic nature and its role in IBD [38]. Across all conditions, stimulation with *F. floriforme* led to an increase in cytokine expression and release. This species induced significantly higher mRNA expression of the pro-inflammatory cytokines interleukin (*IL*) *1β* and *TNFα* throughout all macrophage subsets (Figure 2A,C,E,G,I,K), while a significant increase in TNFα cytokine release was only observed in M1 and M2 macrophages (Figure 2S,W). Additionally, a significant IL1β production was observed in M1 macrophages only (Figure 2Q). Moreover, *IL6* mRNA expression and release were significantly increased in M1 macrophages upon stimulation with *F. floriforme* (Figure 2F,R) while anti-inflammatory *IL10* mRNA expression was significantly expressed in M0 and M2 polarized cells (Figure 2D,L) and a significant increase in the IL10 cytokine level was seen in all macrophage states (Figure 2P,T,X). *C. albicans* only significantly affected *IL1β* and *TNFα* mRNA expression in M1 macrophages (Figure 2E,G) with no effects on the cytokines in all macrophage polarizations (Figure 2M–X). *F. uniguttulatum* did not induce significant elevations of mRNA expression in any of the macrophage subsets (Figure 2A–L). *F. magnum* stimulation resulted in a significant production of IL6 and TNFα in M2 macrophages (Figure 2V–W). Together, this suggests that *Filobasidium* species elicit inflammatory responses in macrophages in mRNA and cytokine level.

### 3.3. Macrophage Cytokine Release after Pre-Treatment with Lipopolysaccharide and Subsequent Stimulation with Filobasidium Species or C. albicans

**Figure 3:**
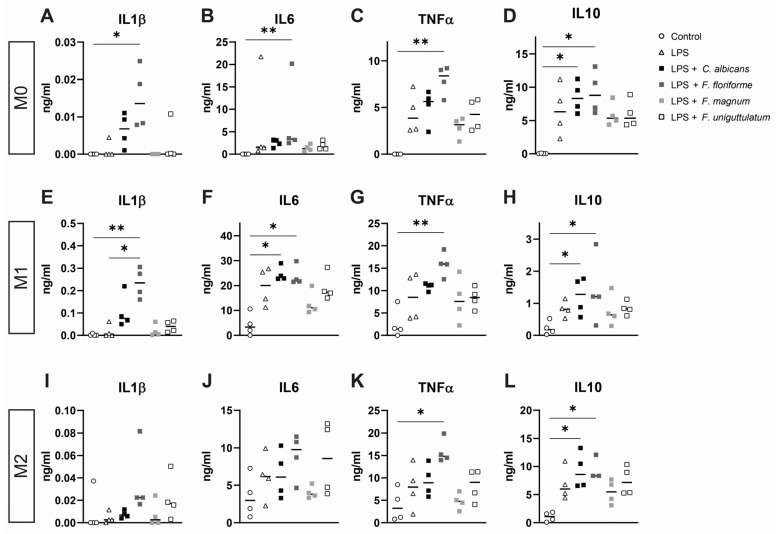
Cytokine release from different states of macrophages after pre-treatment with lipopolysaccharide and stimulation with *Filobasidium* species or *Candida albicans*. (**A**–**L**) Released cytokines by LPS pre-incubated monocyte-derived macrophages skewed into M0, M1, or M2 phenotype ((**A**–**D**), (**E**–**H**), (**I**–**L**), respectively) stimulated with yeasts. All data represented as median and individual data points (*n* = 3–4 donors). * *p* <0.05; ** *p* <0.01.

*Filobasidium* spp. induced a significant stimulation of the expression and secretion of cytokines including IL10 in all conditions tested, which could well explain its association with an improved outcome of FMT. We next tested the effect of pre-incubating macrophages with LPS followed by washing and re-stimulation with three *Filobasidium* species *and C.albicans* spp. Macrophages exposed to *Filobasidium* spp. released significant amounts of TNFα (Figure 3C,G,K), and IL10 (Figure 3D,H,L) in all macrophage sets upon stimulation with *F. floriforme* and LPS. Additionally, IL1β and IL6 were significantly produced in M0 and M1 polarized macrophages upon combined *F. floriforme* and LPS stimulation (Figure 3A,B,E,F). In addition, *Filobasidium* species did not dampen LPS-induced cytokine release in any macrophage state. Interestingly, *C. albicans* significantly affected IL6 cytokine level in M1 macrophages (Figure 3F) and IL10 cytokine production in all macrophage states (Figure 3D,H,L). Concluding, stimulation of macrophages with *F. floriforme*, evokes inflammatory reactions across the board of different macrophage states.

### 3.4. Epithelial Cytokine Release, Gene Expression, and Wound Healing Are Not Affected by Stimulation with Filobasidium spp.

**Figure 4:**
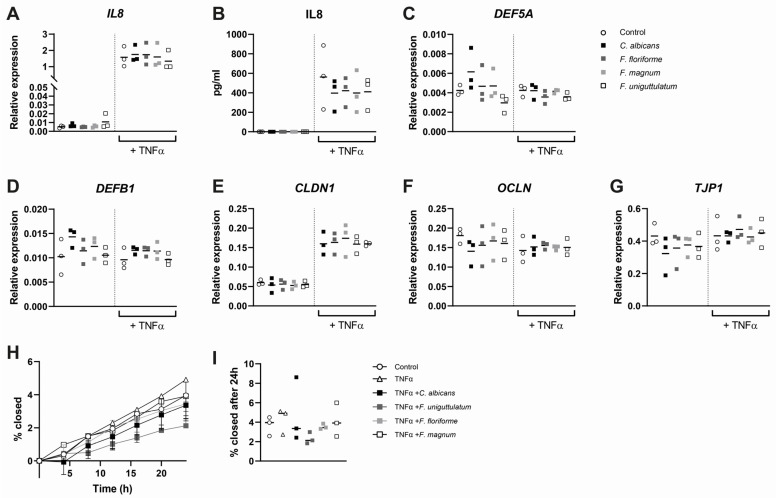
Stimulation of epithelial cells to *Filobasidium* species results in no pro-inflammatory response. (**A**,**C**–**G**) Relative expression of *IL8*, *DEF5A*, *DEFB1*, *CLDN1*, *OCLN*, and *TJP1* upon stimulation of HT29 epithelial cells with yeast for 4 h (*n* = 3). Expression normalized against references genes *HPRT* and *GAPDH.* (**B**) Supernatant IL8 concentrations after 24 h stimulation of HT29 monolayers. (**H**) Epithelial wound healing assay with simultaneous stimulation with yeast species. Closure is expressed as percentage of scratch area closed at 4 h intervals for 24 h. (**I**) Wound closure after 24 h of incubation with yeast. Data are presented as median and separate data points and were tested using Kruskal–Wallis tests.

Given recent insights that yeasts may influence intestinal wound healing [9], we next investigated the effects of stimulation with *Filobasidium* spp. stimulation on epithelial cells. To this end, HT29 monolayers were exposed to the three *Filobasidium* species or *C. albicans* in presence or absence of TNFα to mimic inflammatory conditions. Stimulation of HT29 cells with TNFα stimulated IL8 mRNA expression and cytokine release, while no production of IL8 in absence of TNFα was observed (Figure 4A,B). Furthermore, mRNA expression of the tight junction protein Claudin-1 (*CLDN1*) expression was elevated as well upon TNFα exposure, while antimicrobial peptides (*DEFA5*, *DEFB1*) and other tight junction genes (ZO1 (*TJP1*), Occludin (*OCLN*)) remained unaffected (Figure 4C–G). However, stimulation with the selected yeasts species did not alter expression of any of the assessed genes. We next assessed whether addition of yeasts to an inflammatory condition could enhance wound healing. Monolayers of HT29 were scratched and exposed to TNFα and *Filobasidium* species or *C. albicans*. The wound closure rate was not significantly different between conditions, nor was the closure percentage at 24 h of stimulation (Figure 4H,I). Concluding, *Filobasidium* species do not influence expression of the investigated epithelial genes or functional wound closure in inflammatory conditions.

## 4. Discussion

In these investigations, we searched for markers associating with positive outcome of FMT in ulcerative colitis. Our ITS1-based gut mycobiota analysis confirmed a previously reported association between responsiveness and relatively high pre-FMT level of *Candida* spp. in acceptors. In addition, we observed enhanced presence of the yeast genus *Filobasidium* in fecal matter for transplantation to associate with clinical remission and endoscopic response. While the most abundant ASVs of the genus *Filobasidium* were genetically closest to *Filobasidium magnum*, we also used *Filobasidium uniguttulatum* and *Filobasidium floriforme* spp. in follow-up investigations. Epithelial cells seemed unresponsive to *Filobasidium* spp., but stimulation of monocyte-derived macrophages resulted in a mixed cytokine response that included release of IL10. Although it remains to be established whether *Filobasidium* spp. itself contributes to induction of remission, this study suggests that elevated abundance of the yeast genus *Filobasidium* in fecal material for transplantation associates with successful outcome of FMT in ulcerative colitis.

Using a well-documented, but relatively small, sample set of a previously published FMT study, we aimed to identify fungal biomarkers associating with favorable response in IBD recipients. Our mycobiota focus was inspired by recent preclinical studies indicating that the gut mycobiome affects experimental colitis via host immune recognition of fungi [8,39,40]. In addition, compositional differences were reported when comparing gut mycobiota of patients with IBD to those of healthy subjects. These changes included alterations of the Basidiomycota to Ascomycota (B/A)-ratio, especially in patients with active disease [4]. Despite the small size of our study population, compared to healthy donors, B/A ratio was also raised in our recipients, but did not decrease during remission. Instead, we observed a trend to even further increase that did not reach significance. In line with our results, others also reported no significant changes in B/A ratio upon FMT in patients with ulcerative colitis [6]. In addition to B/A ratios, and most likely due to their pathogenic nature within mucosal surfaces, *Candida* species are frequently studied in relation to intestinal inflammation. Leonardi et al. showed that high pre-FMT relative abundance of *Candida* spp. in ulcerative colitis associated with successful clinical outcome [6]. We also observed high pre-FMT abundance of the genus *Candida* in FMT-D responders, herewith confirming its predictive value, but no post-FMT decline in relative abundance. Based on the latter result one could argue that, although *Candida* spp. can be considered a biomarker for successful FMT, this genus has no functional relevance for disease activity. However, the genus *Candida* contains a few hundred different species, of which only several possess a pathogenic nature [41]. Thus, a shift toward increased presence of less pathogenic species within this genus may still have direct consequences for disease activity.

When searching for additional fungal biomarkers to predict successful FMT, we identified eight genera that were differently abundant when comparing transplant material of responders and non-responders. Three of these genera, *Kazachstania*, *Erythrobasidium* and *Filobasidium*, were enriched in fecal matter for transplantation of responders and thus associated with positive outcome of FMT. While most reports on *Erythrobasidium* relate to plant material, the food-fermenting genus *Kazachstania* was described in relation to gastrointestinal disease. In a rat model of ileal pouch anal anastomosis, low-dose fluconazole treatment resulted in lower fecal abundance of genus *Kazachstania* and aggravated pouchitis [31]. Moreover, *Kazachstania turicensis* was reported as main fecal marker to discriminate between patients with irritable bowel syndrome hypersensitive to colorectal distension and healthy volunteers [42]. Importantly, the genus *Filobasidium* was already described in relation to IBD. Liguori et al. showed that *F. uniguttulatum* associated with non-inflamed mucosa in active Crohn’s disease patients [34]. However, the elevated abundance of *Filobasidium* spp. was only observed at baseline and was completely absent at the 12-week time point. Engrafting of *Filobasidium* spp. within the recipient’s mycobiota is thus unlikely, and hence we next addressed a possible functional role for the genus *Filobasidium* in the induction of remission immediately upon introduction of this yeast. Unfortunately, our ITS1 sequencing approach did not reach the level of resolution needed for *Filobasidium* species identification. Thus, we selected species based on multiple strategies. *Filobasidium magnum* was chosen based on phylogenetic distance, *Filobasidium uniguttulatum* because of previous research [34] and *Filobasidium floriforme* for its capability to grow at 37 °C. Since gut-resident macrophages play a key role in the balancing of tolerogenic and inflammatory responses against intestinal mycobiota [43], we focused on *Filobasidium* spp.-macrophage interactions in our further investigations. Evidently, the in vivo complexity and full spectrum of macrophage functions can never be mimicked by the simplified M0, M1 and M2 approach chosen in this research. Yet, our stimulation assays provided some species-specific evidence for a possible role of IL10. This anti-inflammatory mediator is relevant in maintaining immune homeostasis and involved in host protection from excessive microbiota targeted immune responses [44]. Release of IL10 by M0, M1, and M2 macrophages did not increase following exposure to *C. albicans*, *F. magnum* and *F. uniguttulatum*, whereas all three macrophage subsets showed enhanced mRNA expression and release of IL10 following exposure to *F. floriforme*. These IL10 cytokine release levels were even further enhanced when, prior to yeast stimulations, macrophage subsets were stimulated with LPS. Indeed, augmented IL10 production upon combined pattern recognition receptor activation was reported earlier [44]. Focusing on the presence of two different human macrophage subsets in IBD inflammatory tissue, Bernardo et al. showed enhanced presence of pro-inflammatory monocyte-like cells, but not the IL10-producing macrophage-like cells [45]. Possibly, *Filobasidium* spp. in fecal material for transplantation, helped to restore immune homeostasis by enhancing IL10 production in locally present tolerogenic macrophages. Such local effect of these yeasts on intestinal healing would be reminiscent of a recently published study on the role of *Debaryomyces hansenii*. This yeast, almost exclusively observed in lesional sites, caused delayed wound repair in Crohn’s disease [9]. Consequently, we suggest that opposite, disease alleviating, effects of locally present *Filobasidium* spp. should be explored in future FMT trials. Initial investigations may include the combined in situ assessment of macrophage phenotypes (differentiation markers and cytokine profiles) and *Filobasidium* spp. localization in the gut.

## 5. Conclusions

In this study, we addressed the gut mycobiome in relation to successful FMT. We confirmed earlier observations that linked responsiveness to the enhanced pre-FMT abundance of *Candida* spp. in recipients. Furthermore, in transplanted material, we showed that the high abundance of three genera, *Kazachstania*, *Erythrobasidium,* and *Filobasidium,* associate with a positive outcome. In vitro investigations suggested that the genus *Filobasidium* may enhance the anti-inflammatory phenotype of macrophages. Because our study confirms the possible role of intestinal yeasts in successful FMT, we suggest that future trials should explore the possibility of using fungal biomarkers for donor and recipient selection procedures.

## Figures and Tables

**Table 1:** Patient baseline characteristics stratified for positive response to fecal microbiota transfer.

	Responders	Non-Responders
	*n* = 9	*n* = 22
**FMT-D, *n* (%)**	4 (44)	7 (32)
**Mean age, years (SD)**	46 (12.7)	41 (11.7)
**Male sex, *n* (%)**	3 (33)	11 (50)
**Median disease duration, years (IQR)**	7 (20)	8 (12)
**Extent of disease, *n* (%)**		
E1, proctitis	0 (0)	0 (0)
E2, left-sided	3 (33)	10 (45)
E3, pancolitis	6 (67)	12 (55)
**Concomitant drug treatment, *n* (%)**	6 (67)	17 (77)
Mesalamine oral	6 (67)	14 (64)
Mesalamine rectal	0 (0)	1 (5)
Thiopurines	0 (0)	1 (5)
Systemic corticosteroids (<10 mg)	0 (0)	1 (5)
**Prior anti-TNF therapy, *n* (%)**	0 (0)	1 (5)
**Median SCCAI score at inclusion (IQR)**	8 (5)	8 (3)
**Mayo endoscopic score at inclusion, *n* (%)**		
Mayo 1	2 (22)	2 (9)
Mayo 2	6 (67)	11 (50)
Mayo 3	1 (11)	9 (41)
**Site of disease at inclusion, *n* (%)**		
Rectum only	1 (11)	3 (14)
Left side of colon	7 (78)	13 (59)
Pancolitis	1 (11)	6 (27)

FMT-D, fecal microbiota transfer using donor feces; IQR, interquartile range; SCCAI, Simple Clinical Colitis Activity Index; SD, standard deviation; TNF, tumor necrosis factor.

**Table 2:** Primer sequences for qPCR reactions.

Gene Name	Gene Symbol	5′-Forward Sequence	5′-Reverse Sequence
*Claudin-1*	*CLDN1*	GGCAGATCCAGTGCAAAGTC	TCACTCCCAGGAGGATGC
*Defensin Beta 1*	*DEFB1*	CGCCATGAGAACTTCCTACC	CCACTGCTGACGCAATTGTA
*Defensin Alpha 5*	*DEFA5*	AAGCAGTCTGGGGAAGACAA	CTAGGAAGCTCAGCGACAGC
*Interleukin 1β*	*IL1* *B*	ACCAAACCTCTTCGAGGCAC	AGCCATCATTTCACTGGCGA
*Interleukin 6*	*IL6*	AGTGAGGAACAAGCCAGAGC	GTCAGGGGTGGTTATTGCAT
*Interleukin 8*	*IL8*	AAATTTGGGGTGGAAAGGTT	TCCTGATTTCTGCAGCTCTGT
*Interleukin 10*	*IL10*	GCCACCCTGATGTCTCAGTT	GTGGAGCAGGTGAAGAATGC
*Occludin*	*OCLN*	TTTGTGGGACAAGGAACACA	ATGCCATGGGACTGTCAACT
*ZO-1*	*TJP1*	AGAGCACAGCAATGGAGGAA	GACGTTTCCCCACTCTGAAA
*Tumor Necrosis Factor Alpha*	*TNF*	CCTGCTGCACTTTGGAGTGA	GAGGGTTTGCTACAACATGGG
*Occludin*	*OCLN*	TTTGTGGGACAAGGAACACA	ATGCCATGGGACTGTCAACT
*Glyceraldehyde-30-Phosphate Dehydrogenase*	*GAPDH*	GTCAGTGGTGGACCTGACCT	TGAGCTTGACAAAGTGGTCG
*Hypoxanthine Phosphoribosyltransferase 1*	*HPRT*	CCTGGCGTCGTGATTAGTGAT	AGACGTTCAGTCCTGTCCATAA
*Peptidylprolyl Isomerase A*	*PPIA*	ACGGCGAGCCCTTGG	TTTCTGCTGTCTTTGGGACCT
*Proteasome 20S Subunit Beta 6*	*PSMB6*	ACCTGATGGCGGGAATCAT	ATCATACCCCCCATAGGCACT

## Data Availability

Raw ITS1-sequencing data have been submitted to the ENA database under accession number PRJEB45955.

## References

[1] Actis G.C., Pellicano R., Fagoonee S., Ribaldone D.G. (2019). History of Inflammatory Bowel Diseases. J. Clin. Med..

[2] Qiu P., Ishimoto T., Fu L., Zhang J., Zhang Z., Liu Y. (2022). The Gut Microbiota in Inflammatory Bowel Disease. Front. Cell. Infect. Microbiol..

[3] Caruso R., Lo B.C., Nunez G. (2020). Host-microbiota interactions in inflammatory bowel disease. Nat. Rev. Immunol..

[4] Sokol H., Leducq V., Aschard H., Pham H.P., Jegou S., Landman C., Cohen D., Liguori G., Bourrier A., Nion-Larmurier I. (2017). Fungal microbiota dysbiosis in IBD. Gut.

[5] Imai T., Inoue R., Kawada Y., Morita Y., Inatomi O., Nishida A., Bamba S., Kawahara M., Andoh A. (2019). Characterization of fungal dysbiosis in Japanese patients with inflammatory bowel disease. J. Gastroenterol..

[6] Leonardi I., Paramsothy S., Doron I., Semon A., Kaakoush N.O., Clemente J.C., Faith J.J., Borody T.J., Mitchell H.M., Colombel J.F. (2020). Fungal Trans-kingdom Dynamics Linked to Responsiveness to Fecal Microbiota Transplantation (FMT) Therapy in Ulcerative Colitis. Cell Host Microbe.

[7] Underhill D.M., Braun J. (2022). Fungal microbiome in inflammatory bowel disease: A critical assessment. J. Clin. Investig..

[8] Limon J.J., Tang J., Li D., Wolf A.J., Michelsen K.S., Funari V., Gargus M., Nguyen C., Sharma P., Maymi V.I. (2019). Malassezia Is Associated with Crohn’s Disease and Exacerbates Colitis in Mouse Models. Cell Host Microbe.

[9] Jain U., Ver Heul A.M., Xiong S., Gregory M.H., Demers E.G., Kern J.T., Lai C.W., Muegge B.D., Barisas D.A.G., Leal-Ekman J.S. (2021). Debaryomyces is enriched in Crohn’s disease intestinal tissue and impairs healing in mice. Science.

[10] Costello S.P., Soo W., Bryant R.V., Jairath V., Hart A.L., Andrews J.M. (2017). Systematic review with meta-analysis: Faecal microbiota transplantation for the induction of remission for active ulcerative colitis. Aliment. Pharmacol. Ther..

[11] Wilson B.C., Vatanen T., Cutfield W.S., O’Sullivan J.M. (2019). The Super-Donor Phenomenon in Fecal Microbiota Transplantation. Front. Cell. Infect. Microbiol..

[12] Rossen N.G., Fuentes S., van der Spek M.J., Tijssen J.G., Hartman J.H., Duflou A., Lowenberg M., van den Brink G.R., Mathus-Vliegen E.M., de Vos W.M. (2015). Findings From a Randomized Controlled Trial of Fecal Transplantation for Patients With Ulcerative Colitis. Gastroenterology.

[13] Fuentes S., Rossen N.G., van der Spek M.J., Hartman J.H., Huuskonen L., Korpela K., Salojärvi J., Aalvink S., de Vos W.M., D’Haens G.R. (2017). Microbial shifts and signatures of long-term remission in ulcerative colitis after faecal microbiota transplantation. ISME J..

[14] Rahman S., Davids M., van Hamersveld P.H.P., Welting O., Rahaoui H., Schuren F., Meijer S.L., van den Wijngaard R.M., Hakvoort T.B.M., de Jonge W.J. (2021). Dietary Curdlan Enhances Bifidobacteria and Reduces Intestinal Inflammation in Mice. Nutrients.

[15] Wang Q., Garrity G.M., Tiedje J.M., Cole J.R. (2007). Naive Bayesian classifier for rapid assignment of rRNA sequences into the new bacterial taxonomy. Appl. Environ. Microbiol..

[16] Nilsson R.H., Larsson K.-H., Taylor A.F.S., Bengtsson-Palme J., Jeppesen T.S., Schigel D., Kennedy P., Picard K., Glöckner F.O., Tedersoo L. (2018). The UNITE database for molecular identification of fungi: Handling dark taxa and parallel taxonomic classifications. Nucleic Acids Res..

[17] McMurdie P.J., Holmes S. (2013). phyloseq: An R package for reproducible interactive analysis and graphics of microbiome census data. PLoS ONE.

[18] Bodenhofer U., Bonatesta E., Horejs-Kainrath C., Hochreiter S. (2015). msa: An R package for multiple sequence alignment. Bioinformatics.

[19] Yu G., Smith D.K., Zhu H., Guan Y., Lam T.T.-Y. (2017). ggtree: An r package for visualization and annotation of phylogenetic trees with their covariates and other associated data. Methods Ecol. Evol..

[20] van Thiel I.A.M., Stavrou A.A., de Jong A. (2022). Genetic and phenotypic diversity of fecal Candida albicans strains in irritable bowel syndrome. Sci. Rep..

[21] Repnik U., Knezevic M., Jeras M. (2003). Simple and cost-effective isolation of monocytes from buffy coats. J. Immunol. Methods.

[22] Prins M.M.C., Giugliano F.P., van Roest M., van de Graaf S.F.J., Koelink P.J., Wildenberg M.E. (2021). Thiopurines correct the effects of autophagy impairment on intestinal healing—A potential role for ARHGAP18/RhoA. Dis. Models Mech..

[23] Schneider C.A., Rasband W.S., Eliceiri K.W. (2012). NIH Image to ImageJ: 25 years of image analysis. Nat. Methods.

[24] Vandesompele J., De Preter K., Pattyn F., Poppe B., Van Roy N., De Paepe A., Speleman F. (2002). Accurate normalization of real-time quantitative RT-PCR data by geometric averaging of multiple internal control genes. Genome Biol.

[25] Ruijter J.M., Ramakers C., Hoogaars W.M., Karlen Y., Bakker O., van den Hoff M.J., Moorman A.F. (2009). Amplification efficiency: Linking baseline and bias in the analysis of quantitative PCR data. Nucleic Acids Res..

[26] Von Elm E., Altman D.G., Egger M., Pocock S.J., Gotzsche P.C., Vandenbroucke J.P., STROBE Initiative (2007). The Strengthening the Reporting of Observational Studies in Epidemiology (STROBE) statement: Guidelines for reporting observational studies. Lancet.

[27] Wickham H. (2016). ggplot2: Elegant Graphics for Data Analysis.

[28] Love M.I., Huber W., Anders S. (2014). Moderated estimation of fold change and dispersion for RNA-seq data with DESeq2. Genome Biol..

[29] Bates D., Mächler M., Bolker B., Walker S. (2015). Fitting Linear Mixed-Effects Models Using lme4. J. Stat. Softw..

[30] Oksanen J., Blanchet F.G., Friendly M., Kindt R., Legendre P., McGlinn D., Minchin P.R., O’Hara R.B., Simpson G.L., Solymos P. (2020). Vegan: Community Ecology Package Version 2.5-7.

[31] Zhu F., Feng D., Ding C., Zhang T., Chen J., Yu Z., Zhao L., Xu Y., Zhu W., Gong J. (2020). Fungal Dysbiosis Aggravates Pouchitis in a Rat Model of Ileal Pouch Anal Anastomosis. Inflamm. Bowel Dis..

[32] Heinsbroek S.E., Oei A., Roelofs J.J., Dhawan S., te Velde A., Gordon S., de Jonge W.J. (2012). Genetic deletion of dectin-1 does not affect the course of murine experimental colitis. BMC Gastroenterol..

[33] Chiaro T.R., Soto R., Zac Stephens W., Kubinak J.L., Petersen C., Gogokhia L., Bell R., Delgado J.C., Cox J., Voth W. (2017). A member of the gut mycobiota modulates host purine metabolism exacerbating colitis in mice. Sci. Transl. Med..

[34] Liguori G., Lamas B., Richard M.L., Brandi G., da Costa G., Hoffmann T.W., Di Simone M.P., Calabrese C., Poggioli G., Langella P. (2016). Fungal Dysbiosis in Mucosa-associated Microbiota of Crohn’s Disease Patients. J. Crohn’s Colitis.

[35] Na Y.R., Stakenborg M., Seok S.H., Matteoli G. (2019). Macrophages in intestinal inflammation and resolution: A potential therapeutic target in IBD. Nat. Rev. Gastroenterol. Hepatol..

[36] Snyder R.J., Lantis J., Kirsner R.S., Shah V., Molyneaux M., Carter M.J. (2016). Macrophages: A review of their role in wound healing and their therapeutic use. Wound Repair Regen..

[37] Chikina A.S., Nadalin F., Maurin M., San-Roman M., Thomas-Bonafos T., Li X.V., Lameiras S., Baulande S., Henri S., Malissen B. (2020). Macrophages Maintain Epithelium Integrity by Limiting Fungal Product Absorption. Cell.

[38] Gerard R., Sendid B., Colombel J.F., Poulain D., Jouault T. (2015). An immunological link between *Candida albicans* colonization and Crohn’s disease. Crit. Rev. Microbiol..

[39] Iliev I.D., Funari V.A., Taylor K.D., Nguyen Q., Reyes C.N., Strom S.P., Brown J., Becker C.A., Fleshner P.R., Dubinsky M. (2012). Interactions between commensal fungi and the C-type lectin receptor Dectin-1 influence colitis. Science.

[40] Leonardi I., Li X., Semon A., Li D., Doron I., Putzel G., Bar A., Prieto D., Rescigno M., McGovern D.P.B. (2018). CX3CR1^+^ mononuclear phagocytes control immunity to intestinal fungi. Science.

[41] Kurtzman C.P., Fell J.W., Boekhout T. (2011). The Yeasts: A Taxonomic Study.

[42] Botschuijver S., Roeselers G., Levin E., Jonkers D.M., Welting O., Heinsbroek S.E.M., de Weerd H.H., Boekhout T., Fornai M., Masclee A.A. (2017). Intestinal Fungal Dysbiosis Is Associated With Visceral Hypersensitivity in Patients With Irritable Bowel Syndrome and Rats. Gastroenterology.

[43] Leonardi I., Li X., Iliev I.D. (2018). Macrophage interactions with fungi and bacteria in inflammatory bowel disease. Curr. Opin. Gastroenterol..

[44] Saraiva M., Vieira P., O’Garra A. (2020). Biology and therapeutic potential of interleukin-10. J. Exp. Med..

[45] Bernardo D., Marin A.C., Fernández-Tomé S., Montalban-Arques A., Carrasco A., Tristán E., Ortega-Moreno L., Mora-Gutiérrez I., Díaz-Guerra A., Caminero-Fernández R. (2018). Human intestinal pro-inflammatory CD11c^high^CCR2^+^CX3CR1^+^ macrophages, but not their tolerogenic CD11c^−^CCR2^−^CX3CR1^−^ counterparts, are expanded in inflammatory bowel disease. Mucosal Immunol..

